# Bacteriophage Adherence to Mucus Mediates Preventive Protection against Pathogenic Bacteria

**DOI:** 10.1128/mBio.01984-19

**Published:** 2019-11-19

**Authors:** Gabriel M. F. Almeida, Elina Laanto, Roghaieh Ashrafi, Lotta-Riina Sundberg

**Affiliations:** a Department of Biological and Environmental Science, University of Jyvaskyla, Jyvaskyla, Finland; b Nanoscience Center, University of Jyvaskyla, Jyvaskyla, Finland; c Molecular and Integrative Biosciences Research Programme, Faculty of Biological and Environmental Sciences, University of Helsinki, Helsinki, Finland; University of California, Irvine

**Keywords:** bacterial virulence, bacteriophage therapy, bacteriophages, host-pathogen interactions, mucosal pathogens

## Abstract

Metazoans were proposed to host bacteriophages on their mucosal surfaces in a symbiotic relationship, where phages provide an external immunity against bacterial infections and the metazoans provide phages a medium for interacting with bacteria. However, scarce empirical evidence and model systems have left the phage-mucus interaction poorly understood. Here, we show that phages bind both to porcine mucus and to rainbow trout (Oncorhynchus mykiss) primary mucus, persist up to 7 days in the mucosa, and provide protection against Flavobacterium columnare. Also, exposure to mucus changes the bacterial phenotype by increasing bacterial virulence and susceptibility to phage infections. This trade-off in bacterial virulence reveals ecological benefit of maintaining phages in the metazoan mucosal surfaces. Tests using other phage-bacterium pairs suggest that phage binding to mucus may be widespread in the biosphere, indicating its importance for disease, ecology, and evolution. This phenomenon may have significant potential to be exploited in preventive phage therapy.

## Abstract (precis)

The mucosal surfaces of animals are habitat for microbes, including viruses. Bacteriophages—viruses that infect bacteria—were shown to be able to bind to mucus. This may result in a symbiotic relationship in which phages find bacterial hosts to infect, protecting the mucus-producing animal from bacterial infections in the process. Here, we studied phage binding on mucus and the effect of mucin on phage-bacterium interactions. The significance of our research is in showing that phage adhesion to mucus results in preventive protection against bacterial infections, which will serve as basis for the development of prophylactic phage therapy approaches. Besides, we also reveal that exposure to mucus upregulates bacterial virulence and that this is exploited by phages for infection, adding one additional layer to the metazoan-bacterium-phage biological interactions and ecology. This phenomenon might be widespread in the biosphere and thus crucial for understanding mucosal diseases, their outcome and treatment.

## INTRODUCTION

Mucus is an essential component of the innate immune system, serving as a selective barrier between metazoans and their environments (1). It is a complex, viscoelastic secretion that, in addition to protecting the host, provides a habitat for countless microbes from all three domains of life, as well as viruses. Mucins, the main components of the mucosal matrix, form a polymer-based hydrogel with lubricant and protective properties. The mucin concentration of the mucus affects the mesh size of the matrix, which controls diffusion through it and provides a first line of defense against pathogens (2). Most infections start from the mucosal surfaces, and with over 200 known microorganisms capable of invading the mucus layers, mucosal infections result in mortality and morbidity, exhibiting clinical and economical importance worldwide (3, 4).

Considering the heterogeneous composition of mucosal surfaces, there is a significant gap in knowledge on how its components influence mucosal infections. Previous research has mainly focused on the pathogen, the host immune response *in vitro*, or the effect of the commensal microbiome for mucosal homeostasis (5–7). Moreover, in 2013, another layer of complexity was added to the already convoluted system when the bacteriophage adherence to mucus (BAM) model was proposed (8). This model, based on indirect evidence and *in vitro* testing, proposes an important and so far overlooked symbiosis between metazoans and bacteriophages (phages): Phages would concentrate on mucosal surfaces by weak interactions with mucins, creating a ubiquitous non-host-derived immunity against bacterial invaders during the mucus colonization process. Phage structural proteins possessing Ig-like folds were proposed to be mediators for phage interaction with mucins. A bioinformatic analysis of 246 double-stranded DNA tailed-phage genomes revealed that roughly 25% have proteins with Ig-like folds, all related to the viral structure (9). Indeed, the virus abundance in the surface mucus layer of eels has been shown to be higher than that in surrounding water; the subdiffusive motion of phages on mucus has been better understood; and the spatial structuring of mucosal surfaces has been speculated to have a role in phage replication strategies (10–12). However, the relevance of phage interaction with metazoan mucus for bacterial infections has not been shown in a natural infection model.

Despite the obvious relevance to host-pathogen interactions and ecology, empirical studies exploring the BAM hypothesis have remained scarce. Fish are naturally covered by mucus layers and thus are excellent model organisms for studying phage-mucus interactions and their consequent influences on bacterial infections, especially those that affect mucosal surfaces. Flavobacterium columnare (phylum *Bacteroidetes*, family *Flavobacteriaceae*), the causative agent of columnaris disease, is a major epidermal pathogen in freshwater aquaculture worldwide and responsible for annual losses in the magnitude of millions of dollars (13, 14). Previous studies have indicated that mucus has a positive effect on *F. columnare* growth and virulence-related traits, suggesting that interaction with the mucosal surfaces is important for the bacterial pathogenesis (15–19). Furthermore, as treatment of columnaris disease is based on antibiotics, more information on phages as tools to prevent bacterial infections is needed to reduce antibiotic leakage into the environment (20). While phage therapy seems a promising option against this (21) and many other diseases, detailed information on the interactions of phages and their host bacteria in the mucosal environment is needed.

Here, we test phage adhesion to metazoan mucus and the consequent changes in bacterial disease. Our data provide novel evidence on phage adherence to mucus in a natural setting and possible trade-offs related to mucin-elicited changes in bacterial virulence and phage susceptibility. Due to peculiarities concerning biological interactions, growing *F. columnare* phages in liquid has always been difficult. Exposure of the hosts to mucin made phage growth in liquid possible, facilitating large-scale production of these phages and improving phage isolations. Phage-mucus interactions open promising possibilities for the development of preventive phage therapy approaches for mucosal diseases caused by bacteria in farmed animals and humans.

## RESULTS

### Ig-like domains in phage structural proteins support the suggestion of interaction mechanisms with mucus.

To find a relevant system for studying the interaction between phages, bacteria, and metazoans, we tested the ability of different phages to bind to mucin-containing agar plates. Phage FCL-2 bound preferentially to mucin plates (*P = *0.000001; *F. columnare* host), as did T4 (*P = *0.000005; Escherichia coli host), V46 (*P = *0.001; *Aeromonas* sp. strain B135 host), FL-1 (*P = *0.001, *Flavobacterium* sp. host), and PRD1 (*P = *0.01, Salmonella enterica host), while FLiP (*Flavobacterium* sp. host) did not (see Fig. S1 in the supplemental material). Except for PRD1, all the mucin-binding viruses were tailed phages with proteins containing putative Ig-like domains.

10.1128/mBio.01984-19.2 FIG S1 Phage adhesion to mucin-containing agar plates. Bars represent the number of plaques counted in the control agar plates or agar plates supplemented with 1% purified porcine mucin. The bacterial hosts used were Flavobacterium columnare (FCL-2), *Flavobacterium* sp. strain B183 (FL-1), Salmonella enterica DS88 (PRD1), Escherichia coli (T4), *Flavobacterium* sp. strain B3303 (FLiP), and *Aeromonas* sp. strain B135 (V46). Each data point represents the average of triplicates and their standard deviation. Unpaired *t* tests were used to compare controls and tested conditions (*, *P* < 0.05; **, *P* < 0.001). Download FIG S1, TIF file, 0.06 MB. Copyright © 2019 Almeida et al. 2019 Almeida et al. This content is distributed under the terms of the Creative Commons Attribution 4.0 International license.

Next, we confirmed the presence of Ig-like domains in the mucin-binding phages by bioinformatics. *In silico* reannotation of the FCL-2 (21) genome using HHPred revealed one open reading frame (ORF17: length, 114 amino acids; accession no. YP_009140518) homologous to an N-terminal domain of orf48 in *Lactococcus* phage TP901-1 (probability, 98.94; E value, 4.5E−10), which has been experimentally shown to encode a baseplate protein with an Ig fold (22). The same result was achieved using Phyre2 for homology detection (confidence, 86.5%; coverage, 53%). In the FCL-2 genome, ORF17 (bearing the Ig fold) is located between a putative peptidase and two hypothetical proteins that are then followed by a putative portal protein and capsid proteins. This suggests a structural function for this protein.

The genome sequencing of *Aeromonas* sp. podovirus V46 revealed a putative capsid protein (accession no. MK733234) that was highly similar to phage P22 (HHPred: probability, 100; E value, 1.7E−62), including the telokin-like Ig domain. In addition, the Phyre2 homology recognition resulted in 100% confidence (86% coverage) on the probability that these sequences are homologous. No apparent Ig-like domains were detected in *Flavobacterium* sp. phage FL-1, although a very weak hit to the same N-terminal domain of orf48 in *Lactococcus* phage TP901-1 (probability, 53.25; E value, 100) as in FCL-2 was received.

The two Flavobacterium psychrophilum phages used in the study did not show apparent homology to Ig-like folds in their predicted ORFs, and neither did the structural proteins of the two tailless phages used, FLiP and PRD1.

### The skin mucosa of rainbow trout retains phages for 1 week.

**FIG 1:**
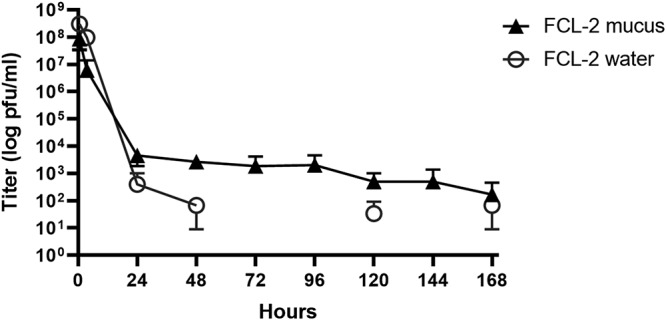
Phages persist on the rainbow trout skin mucosa for up to 1 week after a single exposure. Shown are phage FCL-2 titers in water (gray open circles) and fish mucus (black triangles) over time in a flowthrough aquarium. Fish were exposed to the phage only once, at time zero, and water flow was opened after 3.5 h. Each data point represents the average from three individual fish or three independent water samples and their standard deviation.

Myophage FCL-2, capable of infecting *F. columnare*, was chosen as model for further studies. This phage is well characterized, possess Ig-like domains, and binds to mucins *in vitro*. To verify phage FCL-2 retention by primary mucus *in vivo*, we exposed individual rainbow trout to the phage and kept the fish in flowthrough aquaria. The daily phage titers, in PFU ml^−1^, were measured from the water and fish skin mucus. Phage titers in water decreased rapidly, and at some time points, no phages were detected. However, phages were found on the mucus consistently for up to 7 days after phage exposure, despite the water flow and mucus shedding (Fig. 1). No phages were detected on water or mucus 15 days after exposure (data not shown).

### Pretreatment with phage protects fish from disease.

**FIG 2:**
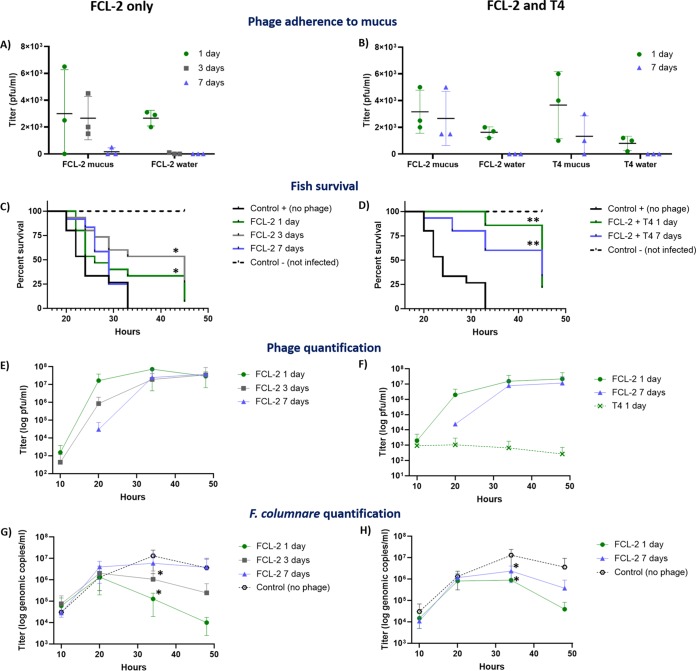
Phage retention on mucus allows for the prophylactic use of phages against bacterial infections. Fish were pretreated with FCL-2 alone (1, 3 or 7 days) or with FCL-2 and T4 (1 or 7 days) before infection with Flavobacterium columnare. (A) FCL-2 phage titers in fish mucus and aquarium water (before infection) from FCL-2-only groups. (B) FCL-2 and T4 titers from fish mucus and aquarium water (before infection) in the combined FCL-2 and T4 groups. (C) Fish survival in the FCL-2-only-pretreated groups. (D) Fish survival in the combined FCL-2- and T4-pretreated groups. (E) FCL-2 titers in water after infection in the FCL-2-only groups. (F) FCL-2 and T4 titers in water after infection in the combined FCL-2 and T4 groups. (G) *F. columnare* quantity (genomic copies) in water after infection in the FCL-2-only-pretreated groups. (H) *F. columnare* quantity (genomic copies) in water after infection in the combined FCL-2- and T4-pretreated groups. Each data point represents one individual fish or water sample and the standard deviations in panels A and B. In panels C and D, the number of fishes used was 15 per group, except in the negative control (*n* = 16) and the group exposed to FCL-2 alone for 7 days (*n* = 12). In panels E, F, G, and H, the data points represent the average of triplicates and their standard deviation. The log-rank (Mantel-Cox) test was used for evaluation of the survival curves in panels C and D. Unpaired *t* tests were used to compare the controls and tested conditions in panels G and H (*, *P* < 0.05; **, *P* < 0.001).

To test if phage retention by mucus is relevant for protection against disease, we exposed rainbow trout to a single dose of FCL-2 phage for 1, 3 or 7 days before exposure to *F. columnare*. To study the influence of a co-occurring phage on FCL-2 mucus adhesion and fish protection, we also exposed the fish to a mixture of FCL-2 and T4 for 1 or 7 days before infection. T4 also contains Ig-like domains and has been shown to bind to mucin, but is not able to infect *F. columnare*. FCL-2 maintained its presence in the mucus of fish from all the preexposed groups, but was found in the water only when exposure occurred 1 or 3 days before infection (Fig. 2A and B). T4 was present in mucus in fish exposed for 1 and 7 days, but only recovered from the water when exposure occurred 1 day before infection (Fig. 2B). The T4 presence did not affect FCL-2 adhesion to mucus, and both phages were recovered at similar titers from the fish.

Exposing the fish to FCL-2 alone or FCL-2 mixed with T4 before the *F. columnare* challenge resulted in a delay in the disease onset and increased fish survival. Fish pretreated with FCL-2 alone for 1 and 3 days survived longer than controls (*P* values of 0.0398 and 0.0013, respectively [Fig. 2C]). When combined with T4, FCL-2 could protect fish in the 1- and 7-day pretreatment groups (*P < *0.0001 and 0.0003, respectively [Fig. 2D]). Interestingly, the presence of T4 enhanced the protective effect of FCL-2, as fish exposed 7 days before infection to both phages had higher survival rates than fish exposed only to FCL-2 in the same time frame (*P = *0.0008).

The FCL-2 titers in the water increased over time, peaking around 35 h after exposure to *F. columnare*, indicating successful replication (Fig. 2E and F). T4 did not replicate in this system. Furthermore, the abundance of *F. columnare* genome copies was measured by quantitative PCR (qPCR) and was inversely proportional to the FCL-2 titers (Fig. 2G and H). Compared with the controls, the reduction of bacterial genome copies was significant at 35 h after infection in the groups pretreated with FCL-2 alone at 1 and 3 days before infection (*P = *0.005 and 0.002, respectively) and groups pretreated with FCL-2 and T4 at 1 and 7 days before infection (*P = *0.004 and 0.009, respectively).

### Exposure to a simulated mucosal environment influences bacterial virulence.

**FIG 3:**
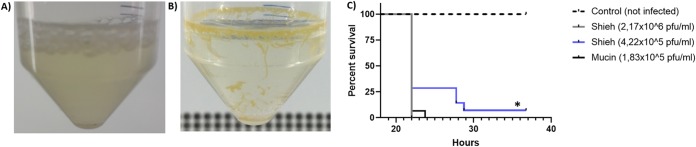
Exposure to mucin increases Flavobacterium columnare virulence. Macroscopic view of a 22-h-old culture of *F. columnare* in 0.5× Shieh medium (A) or in 0.5× Shieh medium supplemented with 0.1% mucin (B). Note the thick biofilm ring on the liquid-air interface, biofilm strings, and clearer supernatant. (C) Fish survival from a virulence test *in vivo* comparing *F. columnare* grown in 0.5× Shieh medium to *F. columnare* grown in 0.5× Shieh medium supplemented with 0.1% mucin. In panel C, the total number of fishes used was 15 per group, except for the group treated with mucin at 1.83 × 10^5^ PFU/ml (*n* = 14). The log-rank (Mantel-Cox) test was used to evaluate survival curves in panel C (*, *P* < 0.05).

In cultures containing mucin as a supplement, *F. columnare* formed a thick biofilm ring on the liquid-air interface and smaller biofilm strings spread over the tube walls (Fig. 3A and B). Details of the biofilm structure can be seen in Fig. S2A to C in the supplemental material. Mucin exposure resulted in positive chemotaxis, larger colony size and improved protease secretion after mucin exposure (Fig. S2D to F).

10.1128/mBio.01984-19.3 FIG S2 Exposure to mucin increases Flavobacterium columnare virulence. (A to C) Helium ion microscopy images of the *F. columnare* mucin-induced biofilm (darker structures) colonizing a block of agar (gray substrate). Scale bars are 20 μm in panel B, 10 μm in panel C, and 5 μm in panel D. (D) *F. columnare* chemotaxis to mucin-containing Shieh medium. (E) *F. columnare* spreading (colony size) on solid Shieh medium containing mucin. (F) *F. columnare* protease halo formed on skim milk-containing plates, in the presence or absence of mucin. Each data point represents the average of duplicates and their standard deviation in panel D, which is representative of two independent experiments. In panels E and F, the data points represents the average of triplicates and their standard deviation. Unpaired *t* tests were used to compare controls and tested conditions on panel E (*, *P* < 0.05; **, *P* < 0.001). Download FIG S2, TIF file, 2.1 MB. Copyright © 2019 Almeida et al. 2019 Almeida et al. This content is distributed under the terms of the Creative Commons Attribution 4.0 International license.

An infection experiment revealed that exposure to mucin increased bacterial virulence *in vivo*. A lower dose of *F. columnare* grown in mucin (11.8 times fewer cells) had the same virulence as a higher dose of cells from control cultures (*P = *0.3137 [Fig. 3C, black and gray lines]). In addition, the cells from mucin cultures killed fish more efficiently than a 2.3 times higher dose from control cultures (*P = *0.0314 [Fig. 3C, black and blue lines]). The survival of fish exposed to control cells was dose dependent (*P = *0.0285 [Fig. 3C, gray and blue lines]).

### Exposure to primary mucus and purified mucin makes bacteria susceptible to phage infection.

**FIG 4:**
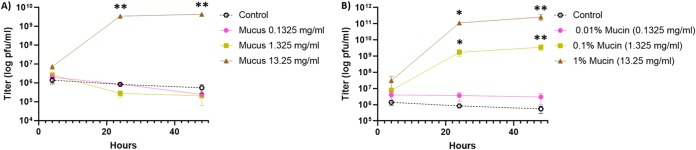
Exposure to simulated mucosal environments makes Flavobacterium columnare susceptible to phage infection. (A) FCL-2 titers over time on 0.5× Shieh cultures supplemented with primary rainbow trout mucus. (B) FCL-2 titers over time on 0.5× Shieh cultures supplemented with purified porcine mucin. Each data point represents the average of triplicates and their standard deviation. Unpaired *t* tests were used to compare the controls and tested conditions (*, *P* < 0.05; **, *P* < 0.001).

Since mucin exposure changes the *F. columnare* phenotypic characteristics, we next studied whether the presence of mucin or mucus affects bacterial susceptibility to phage infections. *F. columnare* strain B185 grown under control conditions or with primary fish mucus or purified porcine mucin was infected with phage FCL-2. The culture aspect in primary mucus cultures was similar to that in mucin-containing cultures (biofilm formation). While the phage yield was negative (i.e., no phage replication) in control cultures and cultures containing low mucus and mucin concentrations, it was hundreds- to thousands fold higher than the initial inoculum at the higher concentrations (Fig. 4A and B). Compared with controls, a significant increase in phage titers was detected 24 and 48 h after infection in media containing 13.25 mg ml^−1^ of mucus (*P < *0.0001), 0.1% mucin (*P = *0.008 and 0.00003, respectively), and 1% mucin (*P = *0.007 and 0.00001, respectively).

Fast biofilm formation and strong biofilm structure make the titration of *F. columnare* cells grown in mucin difficult, so we opted for not quantifying the host cells to avoid misleading comparisons between culture conditions. However, by testing the supernatant of mucin cultures, we were able to determine that phage replication happens in planktonic cells (see Fig. S3A in the supplemental material), and phage presence is able to limit biofilm spread (Fig. S3B). Morphological changes in colony type lasted longer than susceptibility to phages after mucin exposure (see Fig. S4A to C in the supplemental material). Phage FCL-2 growth on other *F. columnare* strains was also favored by mucin exposure, as was growth of other *F. columnare* phages from our collection (see Fig. S5A and B in the supplemental material).

10.1128/mBio.01984-19.4 FIG S3 Phage FCL-2 infects planktonic cells from simulated mucosal cultures and limits biofilm spread. (A) FCL-2 titers on 0.5× Shieh cultures or in 0.5× Shieh cultures supplemented with 0.1% mucin and infected either as a whole (biofilm and plankton together) or divided into biofilm and planktonic cell portions. (B) Qualitative evaluation of the effect of FCL-2 presence on the biofilm spread in mucin-containing cultures. Each data point represents the average of duplicates and their standard deviation in panel A, and the trend was confirmed in an independent experiment. Unpaired *t* tests were used to compare controls and tested conditions (*, *P* < 0.05; **, *P* < 0.001). Download FIG S3, TIF file, 0.7 MB. Copyright © 2019 Almeida et al. 2019 Almeida et al. This content is distributed under the terms of the Creative Commons Attribution 4.0 International license.

10.1128/mBio.01984-19.5 FIG S4 Phage susceptibility induced by simulated mucosal culture exposure disappears faster than morphological colony changes do. (A) Aspect of a new colony morphotype appearing on mucin-containing cultures of Flavobacterium columnare (left). Two conventional rhizoid colonies are evident in the top right. (B) FCL-2 phage titers on sequential passages of cultures on 0.5× Shieh medium and 0.5× Shieh medium supplemented with 0.1% mucin. (C) CFU of *F. columnare*-modified rhizoid colonies from sequential passages of cultures on 0.5× Shieh medium and 0.5× Shieh medium supplemented with 0.1% mucin. Each data point represents the average of triplicates and their standard deviation. Unpaired *t* tests were used to compare controls and tested conditions (**, P < 0.001). Download FIG S4, TIF file, 0.6 MB. Copyright © 2019 Almeida et al. 2019 Almeida et al. This content is distributed under the terms of the Creative Commons Attribution 4.0 International license.

10.1128/mBio.01984-19.6 FIG S5 Effect of exposing Flavobacterium columnare to mucin for phage growth. (A) Phage FCL-2 titers after infections in different *F. columnare* strains grown in 0.5× Shieh medium alone or supplemented with 0.1% mucin. (B) Different *F. columnare* phage (FCOV-F46-62, V156) titers after infection on their respective hosts grown in 0.5× Shieh medium alone or supplemented with 0.1% mucin. Each data point represents the average of triplicates and their standard deviation, except B245 and B350 in panel A and V156 in panel B, which were done in duplicate. Download FIG S5, TIF file, 0.2 MB. Copyright © 2019 Almeida et al. 2019 Almeida et al. This content is distributed under the terms of the Creative Commons Attribution 4.0 International license.

### Changes in bacterial physiology elicited by mucin exposure and the consequent improved phage growth are not limited to *F. columnare*.

**FIG 5:**
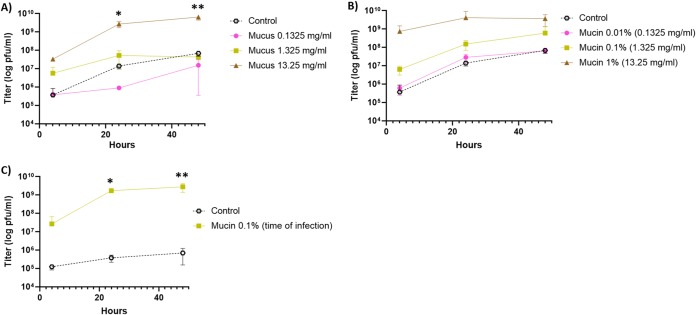
Exposure to simulated mucosal environments makes *Aeromonas* sp. strain B135 susceptible to phage V46. (A) V46 titers over time on 0.2× LB cultures supplemented with primary rainbow trout mucus. (B) V46 titers over time on 0.2× LB cultures supplemented with purified porcine mucin. For panels A and B, the bacteria were grown in mucus or mucin 20 h before infection with phages. (C) V46 titers over time on 0.2× LB cultures supplemented with purified porcine mucin at the time of infection. Each data point represents the average of triplicates and their standard deviation. Unpaired *t* tests were used to compare controls and tested conditions (*, *P* < 0.05; **, *P* < 0.001).

After verifying that mucin exposure changes *F. columnare* virulence and affects phage growth, we tested other bacterial species (see Fig. S6A to F in the supplemental material) to determine the generality of our finding. Cultures supplemented with mucin led to the formation of biofilm in *Aeromonas* sp. strains (B135 and B158), suggesting that mucin exposure also modifies the physiology of these bacteria. Indeed, primary fish mucus made *Aeromonas* sp. strain B135 more susceptible to infection by phage V46 (*P = *0.03 at 24 h and *P = *0.000002 at 48 h [Fig. 5A]). Although no significant effect was initially seen in the purified porcine mucin cultures (Fig. 5B), changing the timing of mucin exposure from preexposure to adding mucin at the time of infection also resulted in improved phage production (*P = *0.03 at 24 h and *P = *0.00006 at 48 h [Fig. 5C]). A clear pattern of higher phage V61 production in *Aeromonas* sp. strain B158 cells exposed to mucin was observed, but phage quantification was not possible due to difficulties in counting the plaques. These data suggest that mucin-mediated changes in the phage-bacterium interaction may be widespread in nature among bacterial species that infect or live on mucosal surfaces.

10.1128/mBio.01984-19.7 FIG S6 Effects of mucin exposure on phage-host interactions in different bacterium-phage pairs. (A) T4 titers after infection in E. coli grown in 0.5× LB alone or supplemented with 1% mucin. (B) PRD1 titers after infection in S. enterica grown in 0.5× LB alone or supplemented with 1% mucin. (C) FL-1 titers after infection in *Flavobacterium* sp. grown in 0.5× Shieh medium alone or supplemented with 1% mucin. (D) FLiP titers after infection in *Flavobacterium* sp. grown in 0.5× Shieh medium alone or supplemented with 1% mucin. (E) FPV4 titers after infection in F. psychrophilum grown in 0.5× TYES alone or supplemented with mucin. (F) FPV9 titers after infection in F. psychrophilum grown in 0.5× TYES alone or supplemented with mucin. Each data point represents the average of triplicates and their standard deviation, except in panels E and F, which were performed in duplicate and confirmed in an independent experiment. Download FIG S6, TIF file, 0.4 MB. Copyright © 2019 Almeida et al. 2019 Almeida et al. This content is distributed under the terms of the Creative Commons Attribution 4.0 International license.

## DISCUSSION

Inspired by the BAM model proposed by Barr et al. (8) and using a natural infection system (rainbow trout, its bacterial pathogen *F. columnare*, and the phage FCL-2), we studied how the mucus environment affects phage-bacterium interactions. We show that several phages bind to mucin, and an *F. columnare*-infecting phage binds to primary fish mucus and purified porcine mucin at comparable levels to those of phage T4. This led us to test the role of phage binding on phage-bacterium interactions and infection in a fish host.

The interaction between mucus and phages has been suggested to occur between Ig-like domains in the phage capsid and glycan residues from mucin molecules. The ORFs in FCL-2 do not show any similarity to the highly antigenic outer capsid protein (hoc) of T4, which mediates adherence to mucus (8). However, FCL-2 ORF17 has homology with a phage protein possessing an Ig-like fold, the lactococcal phage TP901-1 baseplate wedge protein. This implies that the mechanisms for persisting in the mucus for T4 and FCL-2 may be similar. In addition, the *Aeromonas* sp. podovirus V46, which was also enriched in mucin-containing plates, has a highly similar capsid protein to that of phage P22, and both phages possess the telokin-like Ig domain in the capsid protein. The mucus-binding mechanism of phage FL-1 remains speculative. Some of the structural proteins and especially putative tail proteins of FL-1 and FCL-2 are similar, and it is possible that the ORF in FL-1 (length, 134 amino acids; accession no. APZ82894), located between putative capsid and tail genes, has the same function despite clear sequence similarity. The mucin-binding mechanism of the coliphage PRD1, a type of member of the *Tectiviridae*, remains unclear because they do not have distinct Ig-like folds in their structural proteins.

Phage concentrations remained relatively high in the rainbow trout mucus for 1 to 3 days after exposure, providing protection against bacterial infection. A significant delay in the onset of disease and increase in fish survival was obtained, although complete protection was not seen. It is important to note that the experimental conditions with the high dose of bacteria used favored the bacterial infection and not the phage. Interestingly, our results also imply that the co-occurrence with “nonrelevant” phages may influence phage-bacterium interactions in the mucus. Our data suggest that preexposure to FCL-2 combined with T4 protects fish longer than FCL-2 alone, although T4 did not replicate in the system. The mechanism behind this finding is still not known, but since the T4 particle is larger than that of FCL-2, it may help in keeping FCL-2 in the lower mucus layer. More studies are needed to understand how mucus influences the interactions between phages and how combinations of different phages (phage cocktails) may influence protection against infection.

Our results demonstrate that the prophylactic use of phages in phage therapy can be relevant and effective. Exposing metazoans to phages as prophylaxis against bacterial infections has been tested in the past. In 2017, a cocktail of three phages against Vibrio cholerae was shown to be effective if given orally 24 h before infecting mice and rabbits, and phage retention on the intestine was shown (23). In another study, a phage infecting Salmonella enterica serovar Enteritidis was more efficient in protecting poultry if given prophylactically by an oral route. Phages were recovered from feces 7 days after treatment, even in the group not challenged with the bacterial host (24). Furthermore, massive human trials based on preventive phage therapy (called “prophylactic phaging”) were performed in the past by the former Soviet Union. Phage pretreatment reduced disease occurrence, and phage persistence for days in the human body was recorded (25). Although the exact mechanism of phage retention was not studied, it is possible that the success of prophylactic use of phages, under experimental conditions and in human trials, is mediated at least in part by phage subdiffusion on mucosal surfaces. Subdiffusive motion in mucus would hold phages in the mucosa, allowing the phages to find and infect invading mucosal pathogens. A recent finding suggesting mucus layers may negatively affect antibiotic activity on Pseudomonas aeruginosa (26) highlights the potential of phages in protecting against mucosal infections.

In addition to phage retention by mucus, we investigated bacterium and phage interactions on simulated mucosal environments. Culture media containing primary mucus from rainbow trout or purified porcine mucin affected bacterial phenotypic characteristics (see also references 27 and 28) and susceptibility to phage infection. Interestingly, exposure to mucin directly increased bacterial virulence, most likely due to the close relationship of *F. columnare* with its host mucus, demonstrated by the characteristics of the epidermal symptoms of the disease. As such, upregulation of bacterial virulence via mucin exposure may also be relevant for other bacteria, as indicated by two *Aeromonas* sp. strains that form biofilm as a response to primary mucus or mucin. In addition, it has been described that mucin exposure increases the mucus-binding capacity of Lactobacillus reuteri strains, and in this case, mucin may be acting as a signal that changes cell transcriptional status (29).

Furthermore, our results suggest that the mucin-induced upregulation of bacterial virulence has trade-offs in making bacteria more susceptible to phage infection. These data add another component to the bacteria-mucus interaction and the BAM model by suggesting more direct evolutionary benefits of phage binging on mucus by increasing the costs of bacterial virulence. At the molecular level, phages may have evolved to exploit the upregulation of bacterial virulence factors in the mucus environment. This emphasizes the ecological relevance of microbial interactions in the eukaryotic mucus environments, with implications for the outcome of disease. In the case of *F. columnare*, one possible mechanism could be the expression of gliding motility in the presence of mucus. Flavobacterial gliding motility is linked with the type IX secretion system of virulence factors (30), and loss of gliding motility has been associated with a decrease in bacterial virulence and linked with phage resistance for *F. columnare* (31). Indeed, in our study, mucin increased both bacterial colony spreading (indicating increased gliding motility) and secretion of proteases. Increase in motility may increase the availability of surface receptors for phage infections. This is supported by our findings regarding upregulation of bacterial motility by mucin and also by the fact that phages grows preferentially in *F. columnare* planktonic cells. Nevertheless, the mucus-induced changes in bacterial phage susceptibility enabled us to overcome the decades-old difficulties regarding production of *F. columnare* phages in liquid cultures (32). This opens possibilities for isolating new *F. columnare-a*ssociated viruses and growing them for large-scale purposes.

Mucus may have a significant effect for phage–bacterium interactions on a wider scale. A study focusing on Clostridium difficile and phage interaction on human cell cultures concluded that the phage more efficiently reduces the number of planktonic and adherent bacterial cells in the presence of the human cells than occurs *in vitro* (33). Although this suggests that increased phage activity was related to strong phage adsorption to the cells, no data were presented regarding human cells’ effect on the bacterium. The HT-29 cells used are known to be heterogeneous, containing a small percentage of mucin-secreting cells, and HeLa cells produces at least MUC1 (34, 35), so the possibility exists that the released cell-derived mucus changes the bacterial physiology and increases its susceptibility to phages. When testing our collection of freshwater bacteria, we found two *Aeromonas* sp. strains that behaved similarly to *F. columnare* in terms of biofilm formation and phage susceptibility after primary mucus and purified mucin exposure. Although no virulence data exist for these isolates, the *Aeromonas* genus contains many putative disease-causing species (36); thus, interaction with metazoan mucus may also play an important role for these organisms and its interaction with phages.

Our data confirm that phage binding to mucosal surfaces is important for protection against disease. Preferential binding to mucosal surfaces by both bacterial pathogens and their phages suggests that deeper investigation of phage-bacterium interactions in mucus is relevant for understanding mucosal pathogens. This approach provides important mechanistic information on the use of phages for prevention of bacterial diseases and highlights the relevance of the use of “phaging” in future phage therapy trials. Preventive enrichment of the metazoan mucus layers with phages would allow modulation of the host microbiome, thereby generating a protective phage layer that could help to avoid disease. Thus, a prophylactic phage therapy approach could improve the health of farmed animals and also humans. In conclusion, our study has implications that can be applied to provide deeper understanding of the phage-bacterium interactions on metazoan mucosal surfaces on a broader scale.

## MATERIALS AND METHODS

### Bacteria and phages.

The bacterial strains and phages used in this study are listed in Table S1 in the supplemental material. *F. columnare* strain B185 and its phage FCL-2 (myophage) were chosen as models because they have been well characterized and shown to be relevant for phage therapy studies (21). The other strains were used for validation of our observations. Phage lysates were filtered through 0.22-μm-pore filters to obtain cell-free lysates.

10.1128/mBio.01984-19.8 TABLE S1 List of bacterial strains and phages used in this study and their behavior concerning biofilm formation and phage growth on culture media containing purified mucin. Download Table S1, DOCX file, 0.03 MB. Copyright © 2019 Almeida et al. 2019 Almeida et al. This content is distributed under the terms of the Creative Commons Attribution 4.0 International license.

### Growth conditions, phage titrations, primary mucus, and mucin preparation.

*F. columnare*, Flavobacterium johnsoniae, Chryseobacterium indologenes and *Flavobacterium* sp. strains were cultured in modified Shieh medium (without glucose, as described by Song et al. [37]) at 25°C and 120 rpm. F. psychrophilum was cultivated in TYES medium (38) at 15°C and 120 rpm. E. coli and S. enterica were cultivated in LB medium at 37°C and 200 rpm. Aeromonas salmonicida, *Aeromonas* sp. strains, Pseudomonas fluorescens, and Yersinia ruckeri were cultivated in 0.2× LB medium at 25°C and 120 rpm. The phages were titrated using the double-layer agar method on the appropriate hosts (39). Bacterial inocula were normalized by adjusting the optical density at 595 nm (OD_595_) from overnight cultures. In the case of *F. columnare*, a regression curve was used for estimating CFU (CFU from OD values to calculate the inocula and multiplicity of infection [MOI] whenever necessary).

Primary mucus was collected from rainbow trout individuals (Oncorhynchus mykiss) for use in simulated mucosal cultures. Fish (mean size of 8 cm) were euthanized with an overdose of benzocaine, and primary mucus was harvested by scraping the skin with a glass slide. Mucus was pooled on Falcon tubes on ice and briefly centrifuged (1,000 × g, 5 min, 4°C) to remove scales and other debris, and then the supernatant was autoclaved and the sterile mucus was aliquoted and stored at –20°C until use. The total protein concentration was determined by nanodrop measurements. As an example, a batch made from 277 fish yielded around 100 ml of primary mucus with a total protein concentration of 26.5 mg/ml. Alternatively, purified porcine mucin (Sigma, catalog no. M1778) was diluted in water to a 2% final concentration, autoclaved, and stored at 4°C. The total protein concentration of a 2% mucin solution varied from 20 to 26 mg/ml, depending on the batch.

Simulated mucosal cultures were prepared by mixing complete culture media to an equal volume of primary mucus or purified porcine mucin and water, resulting in 0.5× medium containing the desired amount of mucus or mucin. These cultures were made to test how mucin or primary mucus presence would affect biological interactions between phages and bacteria. Doses of 0.1325, 1.325, and 13.25 mg ml^−1^ of primary mucus were used, which corresponded to the total protein concentration of 0.01, 0.1, and 1% purified mucin solutions, respectively. Controls consisted of 0.5× media. Optimal results for *F. columnare* were obtained by inoculating 5 ml of medium with 5 × 10^4^ CFU and then infecting the cultures 22 h later with phages (MOI of 0.01, estimated from the control bacterial number).

### Identification of the Ig-like domain in phage genomes.

The genome of phage FCL-2 has been sequenced (21). Here, the genome was reannotated (September 2018) using protein BLAST (40) and HHPred (41, 42) for protein homology detection and structure prediction. HHPred was also utilized for analyzing ORFs from F. psychrophilum phages FpV-4 and FpV-8, *Salmonella* phage PRD1, and the capsid protein of *Aeromonas* sp. phage V46. In addition, Phyre2 (43) was used for protein fold recognition of predicted structural proteins. Sequences used for the analysis are listed in Table S2 in the supplemental material.

10.1128/mBio.01984-19.9 TABLE S2 Information about the main phages used in this study. Download Table S2, DOCX file, 0.03 MB. Copyright © 2019 Almeida et al. 2019 Almeida et al. This content is distributed under the terms of the Creative Commons Attribution 4.0 International license.

### Phage retention by rainbow trout skin mucus.

To test phage retention in mucosal layers, 30 fish (mean size, 8 ± 0.96 cm) were incubated with 1.25 × 10^12^ PFU of FCL-2 in 2 liters of water (6.25 × 10^8^ PFU ml^−1^) for 30 min. Then, 4 liters of freshwater was added into the aquarium. Three hours later, the water flow was opened, resulting in a change of water at a rate of 860 ml min^−1^ over the duration of the experiment. Aerated water was maintained at 15°C, and the fish were fed daily. At the indicated time points, three fish were collected from the aquarium and euthanized with benzocaine; their skin mucus was scraped with the help of a glass slide and mixed with 450 μl of Shieh medium. Three independent water samples (450 μl each) were also collected. All the samples were preserved by the addition of chloroform (10% final concentration) and used later for phage titrations.

### Prophylactic phage therapy experiment.

The protective effect of phage retention in fish skin mucus was tested. One week before the experiment, 118 rainbow trout (mean size, 8.3 ± 1.12 cm) were moved from the stock tanks to experimental aquaria and acclimatized to 25°C (an increment of +2°C per day, except on the first and last days). At 7 days, 3 days, and 1 day before the infection, designated groups of fishes were exposed to phages. Exposure was made by moving the fish to 4 liters of water containing 1 × 10^12^ PFU of FCL-2 (2.5 × 10^8^ PFU ml^−1^) alone or a mixture of 1 × 10^12^ PFU of FCL-2 and 1.2 × 10^12^ PFU of T4 in 4 liters of water (2.5 × 10^8^ and 3 × 10^8^ PFU ml^−1^, respectively). One aquarium was used for each condition: five for the phage pretreatments and two for controls (fishes not exposed to phages). After 30 min of exposure to phages, the water flow was opened (water flow was 248 ± 35 ml/min). The fish were fed daily until the time of infection and kept in aerated water during all of the experiments.

On the day of infection, the fish were moved to aquaria containing 2 liters of water (each of the seven original aquaria was divided into three aquaria with five fish in each), with no flowthrough. Before the fish were transferred from the phage-treated groups, they were immersed in freshwater to minimize the carryover of phages not attached to the mucus. Then, the bacteria were added to these aquaria for the bacterial immersion challenge (see below), and 2 h later, the fish were moved to larger aquaria containing 6 liters of freshwater (no flowthrough, divisions of three aquaria per tested condition kept). The fish were checked regularly, and dead or moribund fish were removed from the aquaria.

The bacterial inoculum was prepared by adding 2 × 10^6^ CFU of *F. columnare* strain B185 to 200 ml of Shieh medium (triplicates). Twenty-four hours later, at the time of infection, the bacterial number was estimated by OD measurements and confirmed by titrations. The density of bacteria used for infections was 9.96 × 10^8^ PFU per aquarium (4.98 × 10^5^ CFU ml^−1^ in the 2 liters used for the immersion challenge).

The mucus of three fish from each condition tested (phage pretreatments and controls) was collected before the infection process. Water from the original aquaria (where the fishes and phages were kept) was also collected at the same time. At 10, 20, 34, and 48 h after infection, water from all the aquaria was collected. All the samples were preserved by the addition of chloroform to a final concentration of 10% and used for phage titrations and qPCR.

### Virulence *in vivo*.

The effect of mucin exposure to bacterial virulence was tested in an experimental setting similar to that of the prophylactic testing described above but using 59 fish (mean size, 8.08 ± 1.17 cm) and no phage treatments. The bacterial inoculum was prepared by adding 2 × 10^6^ CFU of *F. columnare* strain B185 to control cultures (0.5× Shieh) or 0.5× Shieh supplemented with 0.1% mucin cultures 1 day before the infection. For each condition, three individual 200-ml cultures were prepared. Twenty-four hours later, at the time of infection, the bacterial number was estimated by OD measurements and confirmed by titrations. In the case of mucin cultures, only the supernatant (containing planktonic cells) was used, and the biofilms were discarded. The doses used for infection were 2.17 × 10^6^ and 4.22 × 10^5^ CFU ml^−1^ for controls and 1.83 × 10^5^ CFU ml^−1^ for mucin-grown bacteria in the 2 liters of the immersion challenge. The fish were monitored frequently for disease symptoms and morbidity. Morbid fish that did not respond to external stimuli were considered dead and removed from the experiment.

### Quantitative PCR.

To quantify the *F. columnare* genome copies from aquarium water, a quantitative PCR was designed to detect the CRISPR spacer region in strain B185. The reactions were performed with 5 μl of water samples as the template, 0.3 μM forward (ATTTGGCGGTTGACCATAGAT) and reverse (CGGTGTCCACTTGAAATACCTTAC) primers, and 10 μl of 2× SYBR green Supermix (iQ SYBR green Supermix, Bio-Rad) in a 20-μl reaction volume. All the samples were amplified in triplicate. The PCR started at 95°C for 3 min for initial denaturation, followed by 40 cycles of 15 s at 95°C for denaturation and 60 s at 60°C for annealing. High-resolution melting analysis was performed immediately afterwards by increasing the temperature from 55°C to 95°C by steps of 0.5°C maintained for 5 s each. A standard curve for the quantification of B185 was generated by analyzing a 10-fold dilution series of its DNA with known quantities of 5 ng μl^−1^ to 0.005 pg μl^−1^ 1 (equivalent to 2 × 10^6^ to 2 copies).

### Statistical analysis.

Analysis was done using the GraphPad software version 8.0.1. The log-rank (Mantel-Cox) test was used for evaluation of the survival curves. Unpaired *t* tests were employed for comparison of controls and tested conditions in the appropriate data sets.

### Animal experimentation.

Fish experiments were conducted according to the Finnish Act on the Use of Animals for Experimental Purposes, under permission ESAVI/3940/04.10.07/2015 granted for Lotta-Riina Sundberg by the National Animal Experiment Board at the Regional State Administrative Agency for Southern Finland.

### Data availability.

The data supporting the findings of this study are available within this article and the supplemental material (44–46).

10.1128/mBio.01984-19.1 TEXT S1 Additional experiments concerning phage adhesion to mucin-containing agar, Flavobacterium columnare virulence increase by mucin exposure, and biological interactions between phages and bacteria under simulated mucus conditions. Download Text S1, DOCX file, 0.03 MB. Copyright © 2019 Almeida et al. 2019 Almeida et al. This content is distributed under the terms of the Creative Commons Attribution 4.0 International license.
