# Supplementary material for: Bacteriophage Adherence to Mucus Mediates Preventive Protection against Pathogenic Bacteria
Source: mBio. 2019 Nov 19;10(6):e01984-19. doi: 10.1128/mBio.01984-19 (PMC6867891; doi:10.1128/mBio.01984-19)
Supplement: TABLE S1 [file mBio.01984-19-st001.docx]

**Table S1:** List of bacterial strains and phages used in this study, and their behavior concerning biofilm formation and phage growth on culture media containing purified mucin.

| **Bacterial isolate** | **Growth changes in mucin cultures** | **Tested phages** | **Increased phage yield in mucin cultures** |
| --- | --- | --- | --- |
| *Aeromonas salmonicida^1^* | No | None | - |
| *Aeromonas sp B135^2^* | Yes (biofilm) | V46^2^ | Yes |
| *Aeromonas sp B158^2^* | Yes (biofilm) | V61^2^ | Yes° |
| *Chryseobacterium indologenes^2^* | No | FCL-2^2^ * | No |
| *Escherichia coli* DSM613^3^ | No | T4 DSM4505^3^ | No |
| *F. johnsoniae* UW101*^4^* | No | None | - |
| *Flavobacterium columnare* B067^2^ | Yes (biofilm) | FCL-2^2^ * | No |
| *Flavobacterium columnare* B185^2^ | Yes (biofilm) | FCL-2^2^ , FCOV-F46^2^ , FCOV-F54^2^ , FCOV-F55^2^ | Yes |
| *Flavobacterium columnare* B245^2^ | Yes (biofilm) | FCL-2^2^ *, V156^2^ | Yes for V156 |
| *Flavobacterium columnare* B350^2^ | Yes (biofilm) | FCL-2^2^ * | No |
| *Flavobacterium columnare* B407^2^ | Yes (biofilm) | FCL-2^2^ | Yes |
| *Flavobacterium columnare* B420^2^ | Yes (biofilm) | FCL-2^2^ | Yes |
| *Flavobacterium columnare* B480^2^ | Yes (biofilm) | FCL-2^2^ * | No |
| *Flavobacterium columnare* B537^2^ | Yes (biofilm) | FCOV-F47^2^ , FCOV-F49^2^ , FCOV-F50^2^ , FCOV-F51^2^ , FCOV-F52^2^ , FCOV-F59^2^ , FCOV-F60^2^ , FCOV-F61^2^ , FCOV-F62^2^ | Yes |
| *Flavobacterium columnare* G1^2^ | Yes (biofilm) | FCL-2^2^ | Yes |
| *Flavobacterium columnare* H2^2^ | Yes (biofilm) | FCL-2^2^ * | Yes |
| *Flavobacterium columnare* JIP39/87^5^ | Yes (biofilm) | FCL-2^2^ * , V156^2^ * | No |
| *Flavobacterium columnare* JIP44/87^5^ | Yes (biofilm) | FCL-2^2^ *, V156^2^ * | No |
| *Flavobacterium psychrophilum* 950106-1/1^6^ | No | FPV4^6^ , FPV9^6^ | Negative effect for both with 0.1% mucin |
| *Flavobacterium sp* B183^2^ | No | FL-1^2^ , FCL-2^2^ * | No |
| *Flavobacterium sp* B330^2^ | No | FLiP^2^ | No |
| *Flavobacterium sp* strains B28^2^, B167^2^, B222^2^, B225^2^, B257^2^ | No | None | - |
| *Pseudomonas fluorescens^1^* | No | None | - |
| *Salmonella enterica* Serovar Typhimurium DS88^2^ | No | PRD1^2^ | No |
| *Yersinia ruckeri^1^* | No | None | - |

Origin of the samples: ^1^ Acquired from ATCC. ^2^ Our own collection [**(***46)* and unpublished data]. ^3^ Acquired from DSMZ. ^4^ Kindly donated by Professor Mark McBride (University of Wisconsin-Milwaukee, USA). ^5^ Kindly donated by Professor Jean-Francois Bernardet (INRA, France). ^6^ Kindly donated by Professor Mathias Middelboe (University of Copenhagen, Denmark).

An ***** after a phage name means that it was used to infect a non-susceptible host. ° Although a clear trend was verified, results could not be quantified due to small and turbid plaques.
