# Supplementary material for: Bacteriophage Adherence to Mucus Mediates Preventive Protection against Pathogenic Bacteria
Source: mBio. 2019 Nov 19;10(6):e01984-19. doi: 10.1128/mBio.01984-19 (PMC6867891; doi:10.1128/mBio.01984-19)
Supplement: TABLE S2 [file mBio.01984-19-st002.docx]

Table S2. Information about the main phages used in this study.

| **Phage** | **Accession number** | **Host** | **Tailed** | **Adherence to mucin** | **Increased phage yield in mucin cultures** | **Ig-like domains** | **Carbohydrate binding domains** |
| --- | --- | --- | --- | --- | --- | --- | --- |
| FCL-2 | NC_027125 | *Flavobacterium columnare* | Yes | Yes | Yes | ORF17 homologous to lactococcal phage TP901-1 baseplate wedge protein | None |
| V156 | KY979239 | *Flavobacterium columnare* | Yes | Not tested | Yes | ORF17 identical to ORF17 in FCL-2 | None |
| FL-1 | KY421186 | *Flavobacterium sp* | Yes | Yes | No | Structural proteins similar to FCL-2 | None |
| FLiP | MF361639 | *Flavobacterium sp* | No | No | No | None | None |
| FpV4 | KT876724 | *Flavobacterium psychrophilum* | Yes | Not tested | No | None | Not tested |
| FpV9 | KT876725 | *Flavobacterium psychrophilum* | Yes | Not tested | No | None | Not tested |
| PRD1 | NC_001421.2 | *Salmonella enterica* | No | Yes | No | None | None |
| V46 | MK733234 (capsid protein) | *Aeromonas sp* | Yes | Yes | Yes | Capsid protein homologous to the one of phage P22, with a telokin-like Ig domain | Not tested |
