# Supplementary material for: Bacteriophage Adherence to Mucus Mediates Preventive Protection against Pathogenic Bacteria
Source: mBio. 2019 Nov 19;10(6):e01984-19. doi: 10.1128/mBio.01984-19 (PMC6867891; doi:10.1128/mBio.01984-19)
Supplement: TEXT S1 [file mBio.01984-19-s0001.docx]

Supplementary Material

Phage retention by purified porcine mucin

Five millilitres of media containing phages were added to the top of agar plates supplemented with 1% mucin or without mucin as control. Concentrations of 2.5 x 10^3^ pfu ml^–1^ were used for PRD1, FCL-2, V46, FLiP and FL-1. For T4, 2.5 x 10^2^ pfu ml^–1^ was used due to large plaque sizes. After 30 min of shaking at room temperature, the liquid was removed; the plates were left to dry for 30 min, and then 3 ml of soft agar containing 300 µl of an overnight culture of the bacterial host was added to each plate. The plates were incubated at room temperature, and the plaques were counted 2–3 days later to measure the amount of phages held on the plates. The results are shown in **Fig. S1.** Due to the small plaque size and turbidity of the mucin-containing plates, data on FPV4 or FPV9 (*F. psychrophilum* phages) or V61 (*Aeromonas sp.* B158 phage) was not obtained using this experimental system.

Exposure to a simulated mucosal environment influences bacterial virulence

Bacterial biofilm formed after exposure to purified porcine mucin was imaged by Helium Ion Microscopy. Biofilm samples were prepared by adding a sterile 3 x 3 mm piece of agar to 5 ml of 0.5x Shieh supplemented with 0.1% mucin culture and then inoculating the culture with 5 x 10^4^ cfu of *F. columnare* strain B185. On the next day, the agar piece colonised by the biofilm was fixated and dehydrated, as described in Leppanen et al. *(44)* prior to imaging in a helium ion microscope (Zeiss). Helium ion microscopy imaging of the mucin-generated biofilm revealed that large masses of cells in organised formation growing as independent structures on the colonised surfaces (**Fig. S2A–C**). These formations were absent from control cultures (data not shown).

The influence of mucin on bacterial growth characteristics was seen for all *F. columnare* strains tested (B067, B185, B245, B350, B407, B420, B480, B537, G1, H2, JIP39/87 and JIP44/87), covering genotypes A/C/E/G/H and two reference strains. It was assessed by the appearance of a biofilm ring on the mucin-containing cultures (**Table S1**).

Bacterial attraction to mucin containing solutions was evaluated by chemotaxis assays. The cell density of overnight cultures of *F. columnare* strain B185 was adjusted to 1 x 10^8^ cfu ml^–1^ and 200 µl was added to the wells of a 96-well plate. By using a multichannel pipette (Thermo Scientific, CAT# 46300500), 200 µl of the chemoattractants (consisting of 0.5x Shieh alone or supplemented with mucin) were kept inside the pipette tips and inserted into the wells containing the bacteria. After 90 min, the tip contents were carefully dispensed into clean wells, serially diluted and titrated. The relative chemotaxis response (RCR) was calculated by dividing the cfu count of the tested conditions by the cfu count of the control (0.5x Shieh). Mucin induced positive chemotaxis, with an average relative chemotaxis response (RCR) of 12 to 0.1% mucin and 73 to 1% mucin containing Shieh medium (**Fig. S2D**). This can be taken as a strong chemotactic response, since some authors consider an RCR over 2 to be significant *(45)*.

Colony spread and protease secretion were evaluated in Shieh-agar plates. The cell density of overnight cultures of *F. columnare* strain B185 was adjusted to 1 x 10^6^ cfu ml^–1^, and 10-µl drops were added to the top of Shieh-agar plates supplemented or not with mucin and 1.5% skimmed milk. The colony and protease halo sizes were measured over time in millimetres. Colony spreading of *F. columnare* on agar was also improved by mucin presence (**Fig. S2E**; *p* < 0.0001 comparing controls with agar supplemented with 0.2% or 1% mucin). Furthermore, protease secretion was more prominent on agar plates containing mucin and skimmed milk (**Fig. S2F**).

Phages infect planktonic bacterial cells from mucin cultures.

*F. columnare* B185 mucin cultures (0.1%) were prepared. Before infection with FCL-2, the cultures were either kept as a whole (biofilm and plankton together, as in the previous experiments) or had the supernatant containing planktonic cells transferred to a new flask. Five millilitres of fresh Shieh medium were added to the biofilms left without plankton. All the cultures were infected with the same amount of virus (moi 0.01 based on the cfu estimative of the controls), and the phages were quantified by titration after 4 and 24 h. Phage replication was efficient in cultures containing the planktonic cells from mucin cultures compared to the controls or mucin-generated biofilm alone (**Fig. S3A**). Phage production, measured by dividing the total yield by the inoculum, was negative in the controls and higher in cultures containing only planktonic cells than in cultures containing these cells and the biofilm together.

For evaluating the phage effect on biofilm spread, plastic biofilm baits (used in recirculating aquaculture systems) were employed. *F. columnare* B185 was added to 0.5x Shieh supplemented with 0.1% mucin containing sterile plastic biofilm baits. On the next day, one piece of colonised plastic was transferred to fresh 0.5x Shieh supplemented with 0.1% mucin media, containing more sterile biofilm baits, in the presence or absence of FCL-2. The results were assessed qualitatively by observing the amount of biofilm formed on the flask walls and the sterile biofilm baits 24 h later. This qualitative analysis revealed that the phage presence impairs the ability of *F. columnare* grown in mucin to spread as biofilm to surfaces, but it does not kill biofilm formed before the addition of the phages (**Fig. S3B**). The reduction of biofilm colonisation on the baits and formation of the typical biofilm ring on the mucin culture was phage dose dependent.

The effect of mucin in bacterial physiology persists longer than in phage susceptibility

Due to the fast biofilm formation and strong biofilm structure, attempts to titrate *F. columnare* cells grown in mucin returned variable results. This is caused by difficulties in homogenizing the cultures before making serial dilutions, optical density measurements or nucleic acid extractions. However, from the platings of planktonic cells, a novel colony morphotype was identified (**Fig. S4A**). To understand the duration of the mucin effect on cells, we followed the appearance of this novel colony morphotype and phage susceptibility on liquid over four passages of the original control and mucin cultures. *F. columnare* strain B185 was grown in control (0.5x Shieh) and 0.5x Shieh supplemented with 0.1% mucin cultures. Six cultures were made for each condition, and these cultures were considered to be passage 0. On the next day, 5 µl of each culture was transferred to fresh 0.5x Shieh, and this was called passage 1. Then, half of each of the original tubes (from passage 0) was infected with phage FCL-2. On the next day, the same process was repeated with passage 1 to create passage 2, followed by infection of half the tubes in passage 1. Phages and bacteria from every passage were titrated for 24 h after phage infection. The whole process was repeated until passage 3. The increase in phage titres was taken as a measure of phage susceptibility, while the new colony morphotype was used as a marker for the cell changes elicited by mucin exposure. After an initial exposure to mucin, the cultures were serially passaged daily for 3 days on media without mucin. The same was done for control cultures. Each passage was used for plating planktonic cells, and phage susceptibility was evaluated by infecting the cultures and subsequent quantification of phages by titration after 24 h. Increased phage susceptibility was verified on the original cultures containing mucin (*p* = 0.0003) and the effect disappeared in later passages (**Fig. S4B**). However, the novel colony morphotype was evident in all passages of the mucin cultures, regardless of phage presence, but were absent in the control cultures (**Fig. S4C**).

Host exposure to mucin has a positive effect for all tested *F. columnare* infecting phages, regardless of the host genotype

Mucin exposure had a positive effect for the FCL-2 yield when hosts G1, B407, B420 (G genotypes) and H2 (H genotype) were used. Host H2 is resistant to FCL-2 infections in control cultures, and an effect for the phage host range was seen only in the presence of mucin. However, this was not a broad effect, since no productive infections were observed on hosts B067, B245, B350, JIP39/87 or JIP44/87 (*F. columnare* genotypes A, C and E and two reference strains; **Fig. S5A**). Mucin cultures were also beneficial for other *F. columnare* phages, where 13 phages capable of infecting strains from genotypes C or G from our collection were tested with their respective hosts. Mucin presence had a positive effect for all the C or G genotype phages (FCOV-F47, -F49, -F50, -F51, -F52, -F59, -F60, -F61, -F62, V156 or FCOV-F46, -F54 and -F55, respectively; **Fig. S5B**). It is important to note that all the phages were tested under the same conditions, and adjusting the protocols regarding incubation times, mucin concentrations and removal of biofilms to suit each would probably improve phage replication.

Changes elicited by mucin exposure is linked to mucosal bacteria and not a general feature

When testing the effect of mucin on other bacteria we did not observe changes in bacterial culture phenotype in *Aeromonas salmonicida*, *Escherichia coli*, *Pseudomonas fluorescens*, *Salmonella enterica* or *Yersinia ruckeri* or in the Flavobacteriaceae species *Chryseobacterium* *indologenes*, *Flavobacterium* sp., *F. johnsoniae* or *F. psychrophilum.* Also, mucin presence did not improve the replication of T4, PRD1, FL-1 or FLiP phages in their respective hosts (**Fig. S6A–D**), suggesting that mucins affect only phage binding but not the infection cycle in these phage-bacterium systems. Interestingly, at 0.1%, mucin had a negative effect on the growth of phages FPV4 and FPV9 on *F. psychrophilum* strain 950106-1/1 (**Fig. S6E-F**), a finding that may reveal novel aspects of mucus–bacterium–phage interactions if investigated further.
